# From Mild Cognitive Impairment to Dementia: The Impact of Comorbid Conditions on Disease Conversion

**DOI:** 10.3390/biomedicines12081675

**Published:** 2024-07-26

**Authors:** Federico Menegon, Fabiola De Marchi, Davide Aprile, Iacopo Zanelli, Greta Decaroli, Cristoforo Comi, Giacomo Tondo

**Affiliations:** 1 Neurology Unit, Department of Translational Medicine, Maggiore della Carità Hospital, University of Piemonte Orientale, 28100 Novara, Italy; 20042535@studenti.uniupo.it (F.M.); fabiola.demarchi@uniupo.it (F.D.M.); aprile.davide@hotmail.com (D.A.); 20025584@studenti.uniupo.it (I.Z.); 2 Neurology Unit, Department of Translational Medicine, Sant’Andrea Hospital, University of Piemonte Orientale, Corso Abbiate 21, 13100 Vercelli, Italy; greta.decaroli@gmail.com (G.D.); cristoforo.comi@med.uniupo.it (C.C.); 3 Interdisciplinary Research Center of Autoimmune Diseases (IRCAD), University of Piemonte Orientale, 28100 Novara, Italy

**Keywords:** mild cognitive impairment, dementia, Alzheimer’s disease, conversion, comorbidities, depression, biomarkers, metabolic diseases, disease management, neurodegenerative diseases, vascular dementia

## Abstract

The conversion from mild cognitive impairment (MCI) to dementia is influenced by several factors, including comorbid conditions such as metabolic and vascular diseases. Understanding the impact of these comorbidities can help in the disease management of patients with a higher risk of progressing to dementia, improving outcomes. In the current study, we aimed to analyze data from a large cohort of MCI (n = 188) by principal component analysis (PCA) and cluster analysis (CA) to classify patients into distinct groups based on their comorbidity profile and to predict the risk of conversion to dementia. From our analysis, four clusters emerged. CA showed a significantly higher rate of disease progression for Cluster 1, which was predominantly characterized by extremely high obesity and diabetes compared to other clusters. In contrast, Cluster 3, which was defined by a lower prevalence of all comorbidities, had a lower conversion rate. Cluster 2, mainly including subjects with traumatic brain injuries, showed the lowest rate of conversion. Lastly, Cluster 4, including a high load of hearing loss and depression, showed an intermediate risk of conversion. This study underscores the significant impact of specific comorbidity profiles on the progression from MCI to dementia, highlighting the need for targeted interventions and management strategies for individuals with these comorbidity profiles to potentially delay or prevent the onset of dementia.

## 1. Introduction

Around 50 million people live with dementia worldwide, and this number is projected to almost triplicate by 2050. The number of people with dementia is rising, and it is imperative to identify potentially modifiable risk factors and tackle comorbid conditions since it has been estimated that treating twelve risk factors might prevent up to 40% of dementia [1]. Patients with dementia generally manifest more comorbidities than those without dementia, having an independent impact on outcomes and a consistent socio-economic burden [2]. Several studies reported that comorbidities, including cerebral vascular disease, depression, and chronic obstructive pulmonary disease, may affect the progression of dementia, reducing the quality of life and showing a potential relationship between the underlying disease [3,4,5]. In addition, comorbidities at the time of dementia diagnosis have been associated with decreased survival [6]. Lastly, it has been suggested that treating the comorbid conditions may ameliorate dementia symptoms [7]. Thus, recognizing the importance of comorbidities for dementia care is critical in disease management.

Mild cognitive impairment (MCI) is a heterogeneous condition characterized by a cognitive impairment that does not affect independence in activities of daily living, thus not fulfilling the criteria for dementia. MCI is usually considered a transitional phase between typical aging and dementia. About 10–20% of individuals with MCI annually convert to dementia; thus, a considerable proportion of these individuals will ultimately convert to dementia over 2–5 years [8,9,10,11]. The risk of developing dementia may be affected by concomitant pathologies [1], but the global impact of comorbidities on the conversion from MCI to dementia still needs to be understood. Distinct clinical phenotypes exist, with different risks of progression to dementia, including the amnestic MCI (aMCI) and nonamnestic MCI (naMCI); both include a single domain and a multiple domain subtype [12]. The aMCI is characterized by a prominent or isolated decline in memory. It is usually considered the prodromal phase of Alzheimer’s dementia (AD), despite a not negligible percentage of aMCI subjects remaining stable over time [13,14,15].

The heterogeneity of the MCI condition complicates the precise stratification of subjects according to the expected progression. Identifying MCI subjects at risk for developing dementia is of utmost importance since clinical trials testing disease-modifying therapies should include only participants who will progress to a more severe clinical stage. Research into innovative strategies for managing AD is flourishing, with several therapies currently under investigation [16]. In the last few years, monoclonal antibodies targeting pathological amyloid beta aggregates have been tested in randomized clinical trials, showing an evident effect on clearing beta-amyloid accumulation but without clinical efficacy [17]. There are several reasons behind the unsatisfying results of these trials, including issues related to the participants, since subjects who received the treatments, despite being selected as MCI or mild dementia, could be already in a too advanced stage of the disease, and related to the target, considering the complexity of AD pathology and associated neurodegenerative mechanisms [18]. There is a need in clinical practice and research settings for tools and strategies to recognize patients at risk of developing dementia, even in the preclinical and prodromal stages, to evaluate the efficacy of new possible treatments.

Since its prevalence increases with age, MCI is a condition of the elderly [19]. Coherently, MCI subjects are often affected by multiple comorbidities. In a progressively aging population, an increase in MCI and other comorbidities must be expected. Most of these conditions have been associated with more complicated management, faster development of dementia, or more severe cognitive decline [20,21,22,23,24,25]. Among these conditions, vascular risk factors (VRFs) play a key role in the development of cognitive impairment [26]. In MCI populations, VRF not only contributes to cognitive decline but also promotes conversion to dementia [27]. In addition, the presence of VRF seems associated with the development of multiple-domain impairment, influencing the clinical MCI phenotype [28]. Also, in healthy subjects, VRF participates in neurodegeneration, with an addictive effect on amyloid deposition [29].

Depression is currently considered one of the main risk factors associated with the development of dementia. In particular, late-onset depression, usually involving individuals over 65 years of age, appears to be an accelerating factor for cognitive deterioration [30]. Depression is observed in about 50% of people with AD, and it is directly correlated with the accelerated progression of dementia [31]. In MCI cohorts, depression was shown to affect outcomes and reduce quality of life [32,33]. Depression in MCI contributes to neurodegenerative changes and corresponding clinical impact, being associated with a higher degree of cortical atrophy [34] and with the development of impairment of specific abilities, such as financial capacity [35].

Traumatic brain injury has been recently included, together with alcohol consumption and air pollution, in the group of potentially modifiable risk factors for dementia [1]. Traumatic encephalopathy, which is generally related to chronic sports head injuries, describes a broad range of neuropathology [36]. The history of traumatic brain injury was associated with increased odds of a diagnosis of MCI even after adjusting for age, education, genotype, and VRF. However, the association was attenuated when correcting for the presence of depression, suggesting the need for further confirmation [37].

Self-reported hearing problems are associated with an increased risk of disability and dementia, which is mitigated by the use of hearing aids [38]. Hearing loss is linked with a higher risk of MCI and accelerated cognitive decline [39]. The importance of considering hearing loss as a crucial treatable risk factor for dementia also lies in its vast prevalence since it involves one-third of individuals aged 65 years and older [40].

Preventing, recognizing, and managing comorbidities throughout all stages of life can significantly reduce the risk of developing dementia later in life. This should be one of the most ambitious and promising goals for the global healthcare system. Strategies include not only medications, such as those to reduce blood pressure, but also specific lifestyle modification interventions. The Finnish Geriatric Intervention Study to Prevent Cognitive Impairment and Disability (FINGER) is a multicenter, randomized, controlled study aiming at identifying intervention strategies to prevent the onset of cognitive decline and dementia [41]. The study involved more than one thousand individuals at risk of cognitive decline. Participants underwent a multidomain intervention consisting of diet, exercise, cognitive training, social activity, and control of vascular risk factors. The project demonstrated that administering multidomain interventions is feasible and effective, reporting significant benefits on change in global cognitive performances in older adults at risk for developing dementia [42].

Starting from these premises, several studies explored the prognostic significance of comorbidities in MCI populations, using various statistical approaches and reporting inconsistent results [43,44,45]. A recent study based on the Alzheimer’s Disease Neuroimaging Initiative (ADNI) dataset classified MCI subjects into clinically relevant subtypes according to multiple features, including comorbidities and genetic, imaging, and neuropsychological data, demonstrating the crucial importance of considering comorbidities in the prognostic stratification [46].

This study aimed to identify clinical groups of aMCI subjects with different risks of progression to dementia. We performed a principal component analysis (PCA) and a subsequent cluster analysis (CA) exploring comorbidities in a population referring to the Centres for Dementia and Cognitive Disorders (CDCD) of the University of Piemonte Orientale, Piedmont, Italy, having a diagnosis of aMCI and a suitable follow-up. Subjects were clustered according to comorbid conditions and compared regarding the severity of the progression of cognitive decline and the conversion to dementia, together with other clinical variables, to underline the peculiarity of each cluster. 

## 2. Materials and Methods

### 2.1. Sample Selection

We selected participants whom expert neurologists consecutively evaluated at the CDCD at the Sant’Andrea Hospital, Vercelli, or at the Maggiore della Carità University Hospital, Novara, Italy (University of Piemonte Orientale). We included only subjects aged ≥ 55 years old who were diagnosed with a clinical diagnosis of aMCI using the Petersen criteria [47] from January 2018 to January 2022. All enrolled aMCI subjects had a clinical dementia rating (CDR) scale of 0.5 (questionable dementia), normal cognitive function, and preserved activities of daily living assessed by the evaluation of the Mini-Mental State Examination (MMSE), activity of daily living (ADL), and instrumental activity of daily living (IADL) scores. Cognitive and functional scores (MMSE, ADL, and IADL) were available both at the baseline and the follow-up visits. Since a part of MCI subjects convert to dementia every year, with a variable annual conversion rate of about 10–20%, to avoid bias related to the observational time, other inclusion criteria were considered: (a) symptoms’ onset within three years before the first evaluation (baseline); (b) at least one available follow-up visit between two and five years after the baseline evaluation [48]. We excluded subjects with a diagnosis other than aMCI (naMCI, dementia, psychiatric disturbance, subjective cognitive decline) or with a comorbid neurological condition such as normal pressure hydrocephalus and extrapyramidal syndromes.

### 2.2. Demographic and Clinical Variables

Demographics included age at baseline, sex, educational level, and years from symptoms’ onset. Additional features were familiarity and voluptuary habits, including smoking and alcohol consumption. Among comorbidities, we considered eight recognized AD-related comorbidities [46]: (1) hypertension; (2) diabetes; (3) high cholesterol; (4) depression; (5) obesity; (6) cardiovascular disease; (7) hearing loss; (8) traumatic brain injury (TBI).

We established the presence of depression based on either an anamnestic criterion (reported by the patient or caregiver as a previous diagnosis of depression and with ad hoc questions and in line with the Diagnostic and Statistical Manual of Mental Disorders V criteria) or upon the prescription of antidepressant treatments [49]. For TBI, we based the inclusion on the International Classification of Disease, which defines severe TBI as a skull fracture, edema, brain injury, or bleeding. 

### 2.3. Clinical, Cognitive, and Functional Assessment

Subjects received cognitive and functional assessments using the following scales: MMSE to assess global cognition [50], Katz’s Index of ADL [51], and IADL index [52] to assess the impact of cognitive impairment on everyday living and the correlation between functional and cognitive impairment [53]. For each subject, at the last available follow-up, we collected the established clinical diagnosis, classifying subjects as aMCI (individuals with stable clinical and cognitive status and preserved activities of daily living) and dementia patients (aMCI converted to any type of dementia). Conversion to dementia was considered a binary outcome variable of interest. To assess the degree of cognitive decline over time in each subject, we calculated the index of progression using the formula: follow-up MMSE—baseline MMSE/years of follow-up. The index of progression was used as a continuous variable of interest to compare the degree of cognitive decline over time in different groups of cognitively impaired individuals [54,55]. 

### 2.4. Statistical Analysis

IBM SPSS software (version 25.0, IBM Corporation, Armonk, NY, USA) was used for the statistical analysis, and GraphPad PRISM (Version 9.0, GraphPad Software, Inc., San Diego, CA, USA) was used for the figures. Statistical significance was set at a *p*-value < 0.05.

All continuous data are presented as the mean ± standard deviation (SD). All categorical variables are presented as numbers (percentages). For continuous variables, we used the Kolmogorov–Smirnov test to explore the normality of the distribution of data. Based on the variable distribution of most variables, the ANOVA and Kruskal–Wallis were used for continuous data comparison. A chi-square test was performed to compare categorical variables. PCA was performed to highlight eigenvalues and loading factors. An eigenvalue reflects the amount of variance captured by a given principal component (PC). The eigenvalue-one criterion (eigenvalue ≥ 1) was used to decide how many PCs were to be retained [56]. A factor loading of one independent variable is considered large if its absolute value exceeds 0.45. Then, *k*-means clustering was run to identify discrete clusters within the PCA data, and the obtained clusters were compared to each other. 

We considered as main outcome variables the index of progression, indicating the degree of cognitive decline over time and conversion to dementia at follow-up, identifying two conditions: (1) aMCI and (2) dementia. To investigate comorbid-related clusters associated with conversion to dementia, we conducted a Cox proportional hazards regression model. For each aMCI subject, we considered time 0 as the baseline assessment, the initial event, the diagnosis of aMCI, and the endpoint event, the diagnosis of dementia; the clusters obtained with PCA data were considered covariates. 

This study was approved by the local Ethic Committees (“Comitato Etico Interaziendale Alessandria” and “Comitato Etico Territoriale Interaziendale AOU Maggiore della CArità, Novara”) and performed in compliance with the Declaration of Helsinki.

## 3. Results

### 3.1. Patient Characteristics

The final sample included *n* = 188 participants with the aMCI diagnosis who met the inclusion criteria. Table 1 summarizes the main demographics and clinical and cognitive characteristics of all participants. The median age of participants was 76.00 (IQR: 72.00–79.00), with 91 males (49%) and 97 females (51%). The baseline MMSE score was 25.33 ± 2.60 points; at follow-up, participants scored on average 21.97 ± 4.94 MMSE points, with a mean of −1.13 ± 1.45 points per year lost.

### 3.2. Comorbidities

The mean of comorbidities for each patient in our cohort was 2.00 (SD: 1.31). A total of 118 MCI patients (which corresponds to 63% of the whole cohort) had more than one comorbidity. Table 2 shows the prevalence of the eight considered comorbidities.

### 3.3. Comorbidities PCA

**Figure 1:**
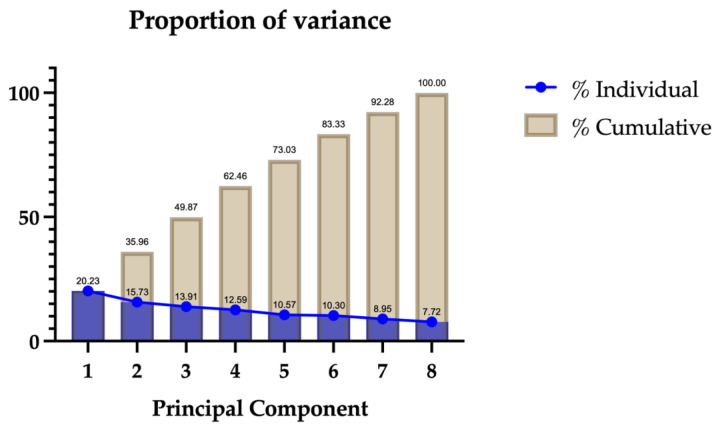
PCA results. Blue: Eigenvalues. Brown: cumulated variance. The *x*-axis represents the principal components, which are the new variables created from the original data. These are ordered by the amount of variance they explain, with PC1 explaining the most variance, followed by PC2, and so on. The *y*-axis represents the proportion of the total variance explained by each principal component. This is shown both individually (for each component) and cumulatively (accumulating the variance explained by all components up to that point). The blue line represents the eigenvalues or the proportion of variance explained by each individual principal component, while the brown bars show the cumulative proportion of variance explained by the principal components.

In this study, we used all comorbidity data to calculate the eigenvalues and the eigenvectors required to obtain the PCs, aiming to determine the main factors for conversion. Since we considered eight factors, eight PCs were generated. Only the eigenvalues of the first four PCs were >1 (1.619, 1.258, 1.112, and 1.007, respectively) and considered for subsequent analysis. These first four PCs explained about 62% of the total variation. Subsequently, with a parallel analysis, only PC1 was selected. PC1 appears to be primarily influenced by factors related to metabolic and cardiovascular health issues, including high cholesterol (loading: −0.663), diabetes (loading: −0.641), cardiovascular diseases (loading: −0.652), and hypertension (loading: −0.551). Other included variables, such as depression (loading: 0.194), hearing loss (loading: −0.029), obesity (loading: −0.026), and TBI (loading: −0.012), have less significant influence on PC1. The eigenvalues obtained and the cumulated variance for each PC are shown in Figure 1. 

### 3.4. Cluster Analysis

From PCA data, we conducted the k-means analysis, obtaining four distinct clusters, which are Cluster 1, Cluster 2, Cluster 3, and Cluster 4. Table 3 presents the final cluster centers derived from *k*-means clustering on standardized PCA scores for various health conditions across four clusters. Each value represents the mean score of the corresponding variable within that cluster. 

**Figure 2:**
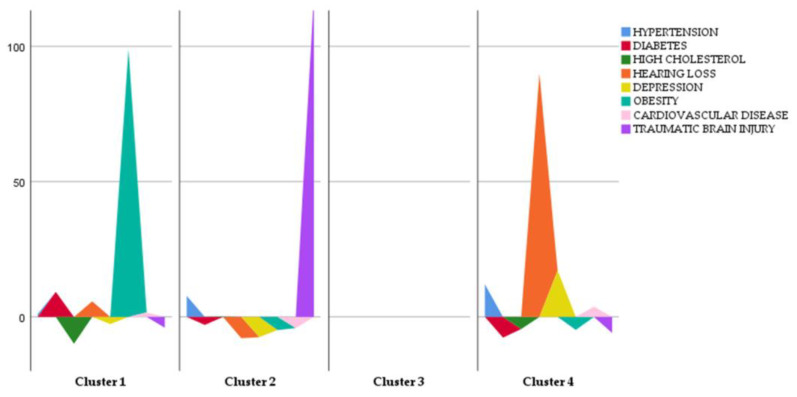
Distinct comorbidity profiles along clusters.

The *k*-means clustering results highlighted distinct profiles within the patient population (Figure 2):

- Cluster 1: predominantly characterized by extremely high obesity and diabetes;
- Cluster 2: marked by a very high TBI;
- Cluster 3: a mixed cluster with slight increases in several conditions but generally lower for others;
- Cluster 4: notable for very high levels of hearing loss and depression.

Table 4 shows a summary of the sociodemographic and clinical features in each of the four clusters. All the clusters had similar average values for age, gender, MMSE at baseline, and follow-up duration. 

When comparing cognitive and functional outcomes, significant differences among clusters emerged. In the whole group, after a mean follow-up of 3.13 years, 98 aMCI subjects (52%) converted to dementia. In Cluster 1, 90% of subjects converted to dementia, while the rate of conversion was 33%, 51%, and 57%, respectively, in Clusters 2, 3, and 4. The Cox proportional hazards regression model showed that cluster 1 had a significant effect on the progression from aMCI to dementia (*p*-value = 0.004). The result is also supported by the rate of the index of progression, where post hoc tests showed that Cluster 1 exhibited a significantly higher index of progression compared to Cluster 2, Cluster 3, and Cluster 4, indicating faster cognitive decline (F = 7.003; *p*-value < 0.001). 

**Figure 3:**
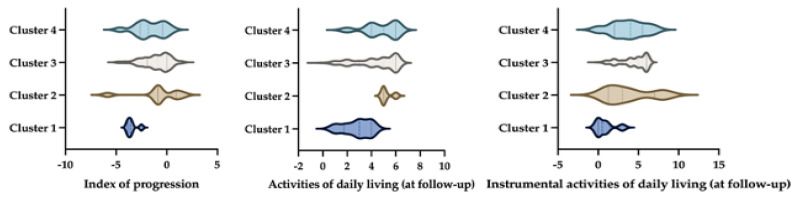
Representation of cluster differences for an index of progression (**left**), activities of daily living (**middle**), and instrumental activities of daily living (**right**). The image illustrates the differences between clusters in three key areas. On the left, it shows an index of progression, which likely measures how dementia progresses over time. In the middle, it presents activities of daily living (ADLs), which include basic self-care tasks. On the right, it displays instrumental activities of daily living (IADLs), which are more complex activities necessary for independent living. By comparing these clusters, the image highlights how different groups vary in terms of disease progression and their ability to perform daily and instrumental activities.

Moreover, regarding functional abilities, at the follow-up, we observed that patients belonging to Cluster 1 had higher ADL/IADL impairments, showing a statistically significant difference in the IADL score when compared with other clusters (F = 7.93, *p*-value < 0.001) and a statistical trend in the ADL score comparison (F = 2.33, *p*-value = 0.057) (see Figure 3). 

## 4. Discussion

Our study investigated the impact of comorbidities on the progression of cognitive decline in a large, amnestic MCI population. We explored eight comorbid conditions recognized to play a role in dementia workup [46]. The PCA stratified MCI subjects according to the weight of each comorbidity, and the subsequent CA allowed us to identify different risk profiles for cognitive impairment progression. Our main results showed that some comorbid conditions associated with high cerebrovascular risk, including obesity and diabetes, have the most critical impact on dementia progression, confirming the importance of considering comorbidities when stratifying subjects with cognitive decline for prognostic considerations.

Substantial evidence testified to the cumulative effect of the association between chronic conditions and dementia, reporting that comorbidities including cardiovascular disease, diabetes, depression, bowel, renal, and respiratory diseases may influence cognitive decline [20,56,57,58]. Consistent with clinical observations, pathological studies confirmed the occurrence of co-pathologies, contributing to a detrimental effect and faster disease progression, steeper cognitive decline, or atypical presentations [22]. In addition, the presence of comorbid conditions may have an impact on the management of patients with cognitive decline and dementia, adding complexity to the therapeutic workup.

Cluster analysis successfully separated individuals showing different conversion risks to dementia and presenting significantly different clinical courses. Cluster 1 was predominantly characterized by a higher weight of two comorbidities, namely obesity and diabetes. Individuals in Cluster 1 had the highest conversion rate and the fastest cognitive decline over time, losing 3.48 MMSE points per year over the considered follow-up. In addition, aMCI in Cluster 1 has suffered from the most important functional decline over time, as revealed by the lowest scores in the ADL and IADL scales. Cluster 2 was dominated by TBI and showed the lowest conversion rate and index of progression despite having the longest follow-up time (3.28 years). Besides TBI, only hypertension, which was equally distributed among clusters, appears to contribute relatively to this cluster. We can thus consider Cluster 2 a particular sample of our aMCI population, characterized by a low weight of comorbid conditions, which is also supported by the youngest age of the group. In addition, although the Lancet Commission [1] considers severe TBI as a significant risk factor for dementia development in the early period after TBI, the pathogenic link remains elusive, and a study involving more than 2500 MCI subjects showed that TBI was not clearly associated with progression to AD over a very long follow-up (8 years) [59]. In line with our results from the Global Burden of Disease Study 2019, the relative risk for dementia in TBI patients was the lowest in the youngest group and decreased across the age range. In addition, considering the strong age gradient, this study reported that less than 2% of the global prevalence of dementia was related to TBI [60].

A slight occurrence of several comorbidities characterized Cluster 3, but without a prominent condition. Lastly, Cluster 4 was characterized by a high prevalence of hearing loss and depression. Individuals in Cluster 4 showed the second most rapid cognitive decline over time among clusters (1.64 points lost per year) and the second highest conversion rate, with 57% of subjects converting after the considered follow-up. 

Our study entails important practical repercussions. In the overall management of individuals with MCI, special efforts should be made to eliminate potentially modifiable risk factors associated with a higher risk of progression of cognitive decline. In our analysis, obesity and diabetes were shown to be associated with a significant risk of conversion. 

Obesity has been associated with the development of cognitive deficits and dementia [61]. Specifically, obesity in midlife is a significant risk factor for developing dementia in old age [62]. In addition, a relationship between obesity and impairment of ADL and IADL has been reported in older adults, suggesting that obese subjects have a higher degree of disability as compared to individuals with typical body weight [63]. However, contrasting results have also been reported [63,64]. A recent study delineated no difference in the rate of decline in global cognition between obese participants and participants with typical weights in a pooled data analysis of 28,867 participants, even with lower baseline cognitive scores in obese individuals [65]. The molecular link between obesity and dementia has been explored in several studies. A pathogenic mechanism linked to hormonal pathways and neuroinflammatory responses has been hypothesized. However, further investigation is needed to reveal the underlying processes [66]. The cross-talk between neuroinflammation, diet, and nutrition has received growing attention due to factors representing easily accessible and modifiable lifestyle components impacting AD and other dementia development [67].

Diabetes is another cerebrovascular risk factor associated with the development of both AD and vascular dementia [68], and diabetic patients have a higher risk of developing cognitive decline than non-diabetic individuals [69,70]. Diabetes is generally associated with accelerated cognitive decline [71]; however, there are also studies reporting no association between diabetes and AD [72,73]. Pathogenic mechanisms related to vascular abnormalities, neuroinflammation, and amyloidosis have been advocated to explain the link between diabetes and cognitive impairment [74], and antidiabetic therapies have been proposed as potential AD therapies [75,76]. Along with the increased risk of cognitive dysfunction, patients with diabetes have an increased risk of functional disability as measured by ADL and IADL impairment compared to subjects without diabetes [77]. Since increased disability reflects diabetic complications and is associated with higher social costs, preventive and therapeutic interventions for metabolic dysfunctions are required in order to achieve healthy brain function.

Cluster 4, showing an intermediate risk of conversion to dementia and mild progression of cognitive decline and functional impairment over time, was characterized by a high load of hearing loss and depression. 

Hearing loss has been suggested as a risk factor for dementia [78,79], and it has been estimated to account for 9% of cases of dementia [1]. The link between hearing loss and dementia has been explained by the impoverished environment associated with hearing impairment, which leads to altered cortical architecture and decreased cognitive reserve [80]. In this vein, hearing screening and treatment may represent a successful strategy for mitigating this risk factor and preventing cognitive decline. 

A history of depression has been related to the development of dementia later in life [22]. The association between depression and AD significantly impacts quality of life and autonomy in activities of daily living [81]. Several studies reported an association between depression and AD; depression may precede the onset of dementia, manifesting already in the preclinical phase and representing the earliest sign of dementia [82,83]. Evidence suggests that antidepressant therapy may influence the development and progression of cognitive decline by stimulating neurogenesis in the hippocampus, modulating neuroinflammatory responses, and inhibiting amyloid deposition [84]. Thus, the recognition of depression is a crucial step in the management of patients with cognitive impairment.

Since MCI and dementia are conditions associated with advanced age, and aging is often associated with other medical conditions and comorbidities, the impact of comorbid conditions on cognitive impairment is not trivial. Comorbidities are often treatable and sometimes reversible; thus, preventive programs are warranted to counter cognitive decline associated with or exacerbated by concomitant conditions. 

We recognize some limitations in our study. The most critical point regards the lack of biomarker inclusion, which would have allowed a more precise subject stratification and a more accurate prognostic prediction. We aimed to identify clinical variables that are easily recognizable and to test their impact on the progression of cognitive decline from the clinical condition of amnestic MCI to dementia. To reduce variability in the outcome, we included only subjects with a follow-up between two and five years. This allowed us to compare cognitive and functional scores between subjects in different clusters. As the results confirmed, the clusters were matched for clinical variables other than comorbidities, including age, educational level, sex, cognitive and functional scores at baseline, and follow-up duration. Thus, we can infer that differences in cognitive decline progression were related to the comorbidity-related classification. However, we used comorbidities as binary variables (presence/absence) without considering the severity of each condition, which would have influenced results, especially functional effects. Further longitudinal analysis with a larger sample and more detailed clinical and biomarker characterization is needed to confirm our data. Lastly, the small sample of some clusters might influence some results; as an example, most subjects belonged to Cluster 3, which was characterized by the presence of several comorbidities but without a prominent condition. It would be interesting to both better stratify participants in Cluster 3 and implement the cohorts included in the other clusters. Despite these limitations, our study provides an original stratification of amnestic MCI subjects related to comorbidities, showing that different clusters have different risk profiles. Our study points out the essential role of comorbidities in contributing to cognitive decline and progression to dementia.

## 5. Conclusions

In the current study, we performed a multi-modal analysis of comorbid conditions in aMCI, delineating clusters of individuals with significantly different clinical progression. The highest risk of cognitive and functional decline over time was observed in the cluster associated with obesity and diabetes, followed by the cluster associated with hearing loss and depression. Identifying aMCI groups with different risk profiles and rates of progression is of utmost importance in prognostic stratification and management of patients with cognitive decline, as well as affecting the design of clinical trials since the inclusion of subjects with mixed pathology might impact the outcomes. This study underlines the importance of preventing comorbid conditions, especially those related to cerebrovascular risk, to counter cognitive decline. 

## Figures and Tables

**Table 1:** Subject demographic, clinical, and cognitive characteristics.

Sample Characteristics	MCI Cohort (n = 188)
Age at the first evaluation (years)	76.00 (IQR: 72.00–79.00)
Male/female	91 (49%)/97 (51%)
Years of education	5.00 (IQR: 5.00–8.00)
Symptoms onset (months)	12.00 (IQR: 6.00–24.00)
Follow-up duration (months)	36.00 (IQR: 30.00–45.00)
Baseline MMSE corrected score	25.33 (SD: 2.60)
Follow-up MMSE corrected score	21.97 (SD: 4.94)
Index of progression (loss of MMSE score/year)	−1.13 (SD: 1.45)

MCI: mild cognitive impairment; IQR: interquartile range; SD: standard deviation; MMSE: mini-mental state examination.

**Table 2:** Prevalence of comorbidities in MCI cohort.

Comorbidity	Number of Patients (%)
Hypertension	120 (64%)
Diabetes	51 (27%)
Hypercholesterolemia	83 (44%)
Hearing loss	15 (8%)
Depression	43 (23%)
Obesity	6 (3%)
Cardiovascular diseases	55 (29%)
Traumatic brain injury	9 (5%)

**Table 3:** Final clusters center for each comorbidity. Each row represents a comorbidity, and each column under “Cluster” represents the center value of that comorbidity within a particular cluster.

Comorbidity	Cluster 1	Cluster 2	Cluster 3	Cluster 4
Hypertension	0.06	0.29	−0.06	0.45
Diabetes	0.51	−0.11	0.01	−0.29
Hypercholesterolemia	−0.55	0.06	0.04	−0.17
Hearing loss	0.32	−0.29	−0.29	3.38
Depression	−0.15	−0.28	−0.04	0.64
Obesity	5.49	−0.18	−0.18	−0.18
Cardiovascular diseases	0.09	−0.15	−0.01	0.14
Traumatic brain injury	−0.22	4.44	−0.22	−0.22

**Table 4:** Cluster sociodemographic characteristics. This table presents the sociodemographic characteristics and clinical data of patients in each cluster identified through the k-means clustering analysis.

Cluster	N. of Cases	Age (Years)	Gender (M/F)	MMSE at Baseline	Follow-Up (Years)	ADL/ IADL	Index of Progression	% of Converters
1	6	79.33	1/5	25.58	2.54	2.83/0.83	−3.48	90
2	9	72.11	4/5	24.62	3.28	5.33/3.75	−0.73	33
3	159	75.83	81/78	25.25	3.16	4.38/3.47	−1.01	51
4	14	76.36	5/9	25.32	2.92	4.78/3.67	−1.64	57

M: male; F: female; MMSE: mini-mental state examination; ADL and IADL are intended for follow-up; *p*-value for index of progression: <0.001; other *p*-values > 0.05.

## Data Availability

Data will be made available on request.
